# Doing Phenomenological Research and Writing

**DOI:** 10.1177/10497323211003058

**Published:** 2021-05-07

**Authors:** Michael van Manen, Max van Manen

**Affiliations:** 1 University of Alberta, Edmonton, Alberta, Canada

**Keywords:** phenomenological publications, the epoché, the reduction, inceptual insights, the phenomenological attitude, the phenomenological example, foundational, exegesis, on the phenomena, the thinging of things, phenomenology of practice, qualitative research

## Abstract

When looking through phenomenology articles in human science and philosophy journals, we may be excused to get the impression that they offer an inconsistent array of phenomenology publications. In this article, we describe three simple but helpful distinctions for determining some order: first, the great foundational publications; second, exegetical publications in the wake of the great works; and third, phenomenological studies done directly on phenomena. Our aim in this article is not to lay claim to phenomenology as a label but rather to discuss how “doing phenomenology directly on the phenomena and the things” means taking up a certain attitude and practicing an attentive awareness to the things of the world as we live and experience them. We propose that engaging in philosophical exegesis and argumentation is not very helpful for analyzing and explicating originary meanings of experiential phenomena. And we show how doing phenomenology directly on the things can be facilitated by a phenomenologically inspired interpretive attitude as well as by a sensitive talent for employing phenomenological examples.

When discussing the subject matter of phenomenology, awkward questions may arise. What kind of phenomenology is most relevant for researchers in fields such as psychology, pedagogy, nursing, and medicine? Are some phenomenological studies more helpful than others for understanding human existence? What do such phenomenological studies look like? One obvious suggestion would be to peruse journals that publish phenomenological articles and in which reputable phenomenologists publish. What kinds of texts and publications do we find with the term “phenomenology” in the title or with the obvious intent to be considered works of phenomenology? One would have to cast the net quite wide, and one would soon discover that some journals, magazines, and other media contain writings of classic authors, specialist historical subjects, treatments of technical philosophical issues, and topics of professional practice in various professional fields, such as psychology, health science, education, pedagogy, technology, and media. In addition, one should not forget that some publications appeal to the spheres of interest of the educated general reader. But no doubt, one will discover that the range of phenomenological publications is quite large and very diverse.

With the intent to provide some order in the plethora of published phenomenological materials, the Husserlian philosopher, Joseph Kockelmans (1987), distinguished three streams of phenomenological publications. These distinctions can still be quite helpful to the reader and are worth considering:

Over the past decades many books and essays have been written on phenomenology. Some of these publications are historical in character and were designed to give the reader an idea of the origin, meaning, and function of phenomenology and its most important trends. Others are theoretical in nature and were written to give the reader an insight into the ways in which various authors conceive of phenomenology and how they attempt to justify their views in light of the philosophical assumptions underlying their conceptions. Finally, there are a great number of publications in which the authors do not talk about phenomenology, but rather try to do what was described as possible and necessary in the first two kinds of publications. (p. vii)

The first stream of publications contains the most original works, of historical relevance, and generally foundational to the field of phenomenology. These are the writings by highly gifted and leading philosophers and human science phenomenologists who developed the original idea of phenomenology, each naming the world in a singular and yet universal nomenclature:

Phenomenology opens onto the excess beyond philosophy from which philosophy draws. Different philosophers name that excess differently: experience, Being, the concrete, the ethical, the trace, and so on. (Bernasconi, 2020, p. 6)

Husserl, Heidegger, Sartre, and Merleau-Ponty are probably among the best known originators, although their work is not always easy to read and comprehend. Still, their original foundational writings offer fundamental insights that appear inexhaustible in their philosophical significance for those seriously interested in phenomenology.

Husserl’s (1954/1970a, 1900–1901/1970b, 1913/1983) works gave us the method of the reduction that must establish the phenomenological attitude, the mode of *intentionality* of consciousness that allows the things of the world to give themselves as phenomena, the epoché that involves the suspension of the *natural attitude* in favor of the *transcendental reduction*, the *lifeworld* as the source of our *lived experiences*, and the means of *bracketing* to assist in identifying eidetic aspects of phenomena. Heidegger’s (1927/1962, 1982, 2001) works gave us the focus on the *Being of being*, human ontology as *Dasein*, the characterization of the phenomenological method as to let that which shows itself be seen from itself in the very way in which it shows itself from itself, his notions of *zuhanden* and *vorhanden*, and his writings on *technology* whereby technology is not to be understood instrumentally but as the explication of the general comportment by which technology may shape our existential ways of being.

Of course, there are other early and subsequent phenomenological publications that offer founding phenomenological ideas, such as in the writings of Jean-Paul Sartre (1956, 1991), Maurice Merleau-Ponty (1962), Max Scheler (1970), Emmanuel Levinas (1979, 1981), and more contemporary originary works of thinkers such as Jean-Luc Nancy (1997, 2007), Jacques Derrida (1995), Michel Henry (1990/2008, 1988/2009), and Jean-Luc Marion (2002, 2007; see van Manen, 2014). These works are indeed recognized as brilliant, original, and pathbreaking texts offering convergent and divergent paths of thought.

As for the second stream that Kockelmans distinguishes, there is the broad scholarly literature that continues to address and explore technical, historical, and theoretical issues of phenomenology. These are publications that tend to take up in an exegetical, critical, and philosophical manner the arguments and positions of other philosophers and scholars of phenomenology. This literature is enormously variegated and extensive, sometimes offering interesting comparative studies and probing thought-provoking topics, and at other times texts that are steeped in “language” and only of interest and readable by specialized exegetical philosophers. The etymology of the term “exegesis” borrows from Latin and Greek, meaning exposition, narrative, and explanation. Exegetical phenomenology tends to be meta-phenomenology. How to recognize this form of phenomenological publications? The general style of these publications is that they offer explanations *of*, theories *about*, comments *on*, and introductions *to* other published phenomenological works, topics, and concerns that tend to be technical and/or historical in a philosophical or specialized disciplinary phenomenological sense.

Kockelmans’s third stream of phenomenological publications is neither primarily presenting new phenomenological foundations nor presenting arguments or developing theories *about* phenomenology and technical philosophical issues and themes. Instead, the third stream of publications is composed of phenomenological texts that actually practice or “do” phenomenology on concrete topics of the lifeworld. They try to do, as Kockelmans says, what was described as possible and necessary in the foundational and theoretical writings of phenomenologists. They “do” what the works of the two streams of founding originators and subsequent commentators suggest or imply are the possible, original, and necessary task of phenomenology: to explicate the originary meaning of the phenomenality of phenomena as they give themselves *in* and *as* human consciousness and experience. Examples of this third stream of phenomenological writings are presented in the 2021 book, *Classic Writings for a Phenomenology of Practice* (van Manen & van Manen). This text contains phenomenological essays and studies exploring and explicating down-to-earth phenomenological research questions, such as the following: How do we encounter a conversation? What is the meaning and significance of secrets in children’s lives? How do we come to experience humor? What is the meaning of “things” in the world of the child? How do we experience obsessive compulsions? And how do we experience a baby’s first smile?

The perceived relevance of the various titles and themes of the third stream of phenomenological publications is obviously dependent on one’s personal life interests, clinical practice, or research project. But the point is that these publications are examples of “doing phenomenology” in a manner that should not be confused with philosophizing about phenomenology or doing primarily exegesis, however interesting that may be in its own right. A philosopher who primarily pursues technical, historical, or other exegetical topics may have little propensity to pursue foundational scholarly issues that are fundamental to the direction of the field or contribute to relevant phenomenological studies that are performed directly on the concrete phenomena and events of everyday and professional life.

To do phenomenology *on* the things, we must turn *to* the things. Maurice Merleau-Ponty (1962) says that this means that we must begin by reawakening the basic experience of the world and by practicing a “direct description” of this world:

[A]ll the efforts [of phenomenology] are concentrated upon re-achieving a direct and primitive contact with the world . . . it also offers an account of space, time and the world as we “live” them. It tries to give a direct description of our experience as it is, without taking into account of its psychological origin and the causal explanations which the scientist, the historian or the sociologist may be able to provide. (p. vii)

Giving a “direct description” of experience is not just narratively reporting, copying, or telling a story. Rather, to de-scribe is to write directly (unravel or uncover) what remained hidden or concealed. Doing phenomenology on the phenomena means taking up the attitude of immediate seeing and practicing an attentive awareness to the things of the world as we live them rather than as we conceptualize or theorize them. Direct description is making straight sense of the originary meanings of lived or inceptual experience (the primal phenomena and events as given in or as consciousness).

## Doing Phenomenology on the Things

Developing phenomenological insights into human existence may be considered the original and primary task of phenomenology. In the contemporary phenomenological literature, these are those phenomenological publications on topics that are meant to be of interest and relevance to ordinary and extraordinary life situations.

Contemporary phenomenological philosophers have produced publications that exemplify doing phenomenology on the phenomena: *The Glance* by Edward Casey (2007), *Abuses* by Alphonso Lingis (2001), *The Thinking Hand* by Juhani Pallasma (2009), *The Fall of Sleep* by Jean-Luc Nancy (2007), *The Five Senses of Veils, Boxes, Tables, Visit, Joy* by Michel Serres (2008), and other studies that offer surprising and fascinating phenomenological insights into the meaning of concrete everyday human experiences and lifeworld events. In addition, there are publications that comprise foundational and exegetical works. For example, a text such as Derrida’s (2005) *On Touching—Jean-Luc Nancy*, is a genre of phenomenological philosophical thinking that transposes the apparent exegetical style of interpreting Jean-Luc Nancy’s texts to a level of originality and fascination that does not only clarify but also (re)invents.

Yet this third stream of phenomenological publications “done directly on the things” is actually rarer than exegetical texts dealing with phenomenology at a level more removed from the concrete reality of human experience. Furthermore, ironically, the third kind of publications are more likely produced by clinical psychologists, psychiatrists, medical doctors, pedagogues, and other professionals who were or are also researchers and university academics.

Historically, one early development of phenomenology on the things came to be known as “the Utrecht School” or “the Dutch School” of phenomenology although some of the authors were German or wrote in French or other languages. The Utrecht studies were probably among the first to focus on actually doing phenomenology on ordinary and professional phenomena. We believe that these writings are challenging and demanding not only because of their scholarly resourcefulness, but also because of the required talents for perceptive phenomenological insights of these early leading proponents.

Some of the well-known topics that were explored as books and as manuscripts by the proponents of the so-called Utrecht School of phenomenology were “The Hotel Room” (David van Lennep, 1953), “Aspects of Sexual Incarnation” (Johannes Linschoten, 1953), “The Psychology of Driving a Car” (David van Lennep, 1953), “The Sickbed” (Jan van den Berg, 1966), “Falling Asleep” (Johannes Linschoten, 1987), “The Human Touch” (Frederik Buytendijk, 1970), and so on. It is not difficult to discern the difference between exegetical phenomenological publications and the studies that may be termed “phenomenology of practice” (in the sense of professional and everyday life phenomena and events).

The work of the authors of these phenomenological writings is unique in that it speaks to the practice of doing phenomenological research to better understand aspects of professional life. The first mention of “the Utrecht School” is probably on the back cover of *Persoon en Wereld* [*Person and World*] (Langeveld, 1953), edited by van den Berg and Linschoten. They stated, “one could say that in the fifties at Utrecht University, a phenomenological school had emerged under the leadership of F. J. J. Buytendijk.” That is likely when and where the title “the Utrecht School” of phenomenology was coined. Van den Berg and Linschoten (1953) further declared programmatically that the phenomenologist resolves to stay as close as possible to the everyday life’s ordinary events. Indeed, these phenomenologists were driven by a quotidian interest in ordinary life phenomena, even as these topics often were born in the contexts of professional practices.

In *Classic Writings for a Phenomenology of Practice*, Michael van Manen and Max van Manen (2021) discuss the Utrecht School of phenomenology as one of the early examples of doing phenomenology directly on the concrete phenomena and events, and reflect on methodological terms in a broad yet specifying sense, such as the phenomenological *attitude* and *example*, that are crucial for phenomenological inquiry and research. “Phenomenology is a method; it could be called an attitude,” said van den Berg (1972, p. 77). But in what sense could the method be called a phenomenological attitude? We show that this attitude consists of a certain way of seeing, thinking, and expressing aimed at *eidetic* (essential) and inceptual insights into the phenomena and events of our existential lifeworld.

Herbert Spiegelberg (1960), the encyclopedic scholar who wrote the authoritative international study entitled *The Phenomenological Movement, A Historical Introduction*, initially scarcely mentioned the early Utrecht School initiatives in his accounts of phenomenological developments. In this two-volume work, he only included the contributions of professional academic philosophers. And none of the Utrecht proponents started out as philosophers. But in his 1972 book, *Phenomenology in Psychology and Psychiatry*, Spiegelberg dedicates an extensive chapter to Frederik J. J. Buytendijk. He described Buytendijk as the “central pioneer” of the Utrecht School movement (p. 281). Buytendijk was a medically trained physician with a research interest in physiology. He received university appointments in medicine, physiology, and psychology as he gained an international reputation for his academic and clinical scholarship.

Herbert Spiegelberg had become famous for his encyclopedic presentations of phenomenological developments around the world. However, by 1975, he had apparently become dissatisfied with the way that phenomenology was progressing and practiced in philosophy. Fifteen years after the first edition of his authoritative *The Phenomenological Movement*, Spiegelberg (1975) published *Doing Phenomenology: Essays On and In Phenomenology*, in which he decried “the relative sterility in phenomenological philosophy . . . especially in comparison with what happened in such countries as France and The Netherlands” (p. 25). He proposed that what was needed is “a revival of the spirit of doing phenomenology directly on the phenomena, the ‘things,’” and he spoke nostalgically of “the spirit which permeated the first generation of phenomenologists” (p. 25).

Spiegelberg asked, “What can be done to reawaken [this spirit] in a very different setting?” (p. 25). He advocated a reorientation of “doing phenomenology on the phenomena themselves” (p. xiv) and he urged “a fresh approach directly to the phenomena in opposition to mere meta-phenomenology through textual and historical studies” (pp. 24, 25). Spiegelberg notes how Husserl had longed for some kind of practice of joint-phenomenologizing. However, Husserl’s seminars were usually dominated by his philosophical monologues. So, Spiegelberg spoke perhaps somewhat mockingly of the philosophic practice of “meta-meta-phenomenology” where “phenomenology can become a flight from the phenomena” themselves, that is, cut off from the intentionality of the experiential lifeworld that was supposedly the focus of phenomenology (p. 22). But, of course, we must realize that exegetical and epistemological explorations of phenomenology can be critical and supportive of the project of doing phenomenological research and writing. Our discussion of the phenomenological attitude, the methodological notion of example, and the idea of a phenomenology of practice are obviously presented at a meta-level in relation to the down-to-earth work of doing phenomenology directly on the things.

In his book *Doing Phenomenology*, Spiegelberg (1975) reported on a pilot experiment of a summer workshop organized as a cooperative phenomenology seminar between 1965 and 1972. He described gradually coming to the idea of a phenomenology workshop, in which phenomenology would be “done” and not just talked about (p. 26). But Spiegelberg was frustrated by the poor results of these workshops that did not seem to yield concrete lifeworld studies. Significantly, similar efforts were meanwhile on the way in the Netherlands—witness the works of Beets, Linschoten, and Beekman, who were students of Langeveld, Buytendijk, and van den Berg. As well, there were the subsequent phenomenology workshops by Beekman at the University of Utrecht. Apparently, Spiegelberg had become aware of the Dutch phenomenology developments and mentions them in his book *Doing Phenomenology*. Ironically, by the time Spiegelberg pointed at these developments, most of the leading figures had retired in the Netherlands and, by the mid-1970s, these phenomenological initiatives had eroded under the pressure of behavioral and empirical analytic science influences from the United Kingdom and the United States.

It is quite remarkable that the philosopher Herbert Spiegelberg initially ignored phenomenological initiatives by scholars who were not professional philosophers themselves. But Spiegelberg later deliberately turned toward scholars in professional fields (rather than to professional philosophers) in providing examples where the “spirit” of doing phenomenology was alive. Indeed, phenomenologists like Buytendijk, van den Berg, Linschoten, Langeveld, and others were guided by a phenomenological way of seeing while doing phenomenology on the phenomena.

Someone can be occupied with writing scholarly articles and books *about* phenomenology at a meta-level or, as Spiegelberg wrote, at a meta-meta-level twice removed from the effort of actually doing phenomenology on the things as intended by Husserl and in his famous dictum “back to the things themselves” (*zu den Sachen selbst*). But writing about phenomenology is not the same as *doing phenomenology directly on phenomena themselves*. The difference is that one can “argue” philosophically about exegetical phenomenological issues and aim to develop philosophical systems while being purblind to phenomenological “seeing” and failing to demonstrate a genuine phenomenological attitude, able to explicate sensitively and insightfully originary meanings of selected lifeworld phenomena. Significantly, in the opening pages to his “Phenomenology as a Rigorous Science,” Husserl (1965, p. 75, 1980, p. 47) makes clear that he is not interested in building some “‘system’ for which we yearn, which is supposed to gleam as an ideal before us in the lowlands where we are doing our investigative work.” It is unfortunate that not more contemporary philosophers seek to pursue their phenomenological interests in the lower (concrete) regions of investigative work that should make our lives more thoughtfully livable.

So, in this article, we are attentive to Spiegelberg’s (1975) phrase “doing phenomenology on the phenomena themselves” to describe the third stream of phenomenological writings that Kockelmans (1987) had identified in his *Phenomenological Psychology: The Dutch School* in which he collected several phenomenological essays. When Spiegelberg recommends doing phenomenology directly on the phenomena, he means not just any phenomena, but “phenomena” and “things” as they give themselves while seen under the spell of a phenomenological attitude. This is what it means to do phenomenology directly on the things, on concrete lived human experiences that are now approached with a sense of wonder regarding their phenomenality. We wonder, what really is the phenomenological meaning of “having a conversation,” “feeling compelled to do something,” “encountering humour,” “experiencing a secret place”? (see van Manen & van Manen, 2021). To approach any such topic as a phenomenon is part of the original intent of doing phenomenology.

The uniqueness of the writings of the Utrecht phenomenologists from the early 1930s to the late 1960s is that these protagonists had a dual interest: their (clinical) professional practice and their enthusiasm for phenomenology. They found in the leading phenomenologists of their time a source for deepened understandings and epiphanic insights of the meaning dimensions of their practices. Van den Berg, Buytendijk, and Langeveld had visited and maintained correspondences with Husserl, Heidegger, Binswanger, Scheler, Sartre, Minkowski, and Merleau-Ponty, and they were very familiar with phenomenological developments.

## “Things” in the World of the Child

In this section, we use some exemplary selected extracts from Langeveld’s phenomenological study “The Thing in the World of the Child.” The text is published in full and discussed in *Classic Writings for a Phenomenology of Practice* by Michael van Manen and Max van Manen (2021).

Nothing seems so clearly, so self-evidently, given as the “things,” the stuff or objects of our world. Children apparently need only to learn what the things are, and in this way, something that was originally strange becomes familiar. This “strange” thing, however, continues to be the same. From the start, a chair is a chair, and it emerges, through the developmental encounters of the child, as an experienced and familiar chair. (Langeveld in van Manen & van Manen, 2021, p. 126)

Langeveld explores a seemingly simple question: How do young children begin to experience things in their lifeworlds? To explore the phenomenological meaning of things in children’s lives, Langeveld uses observations, interactions, and insights from the literature of child development. The way things give themselves in the child’s world is in part determined by the child’s situatedness, physical fitness, adult care, and the qualities of the space and time that condition the child’s world. Langeveld uses the examples of the pencil, the slipper, the gift, the cardboard box, the seesaw, the ball, stones, flowers, and housewares as concrete “things” and examines the meaning of such things through the child’s experiential world.

We appreciate the enigmatic quality of things in the child world. A thing is truly neither just any-thing nor a no-thing. But perhaps it is exactly this multiplicity of plain connotations that reveals the elusive significance of the thingness of things for children. The online Oxford English Dictionary, etymologically defines a “thing” as “some-thing not specified by name,” but it is specifically in the unnamedness where the enigma, strangeness, and otherness of the thingness of the thing resides.

Now, it is tempting to be reminded of Heidegger’s well-known phenomenology of “The Thing,” first published as “Das Ding” in 1951 as a lecture given at the *Bayerischen Akademie der Schönen Kunste*. However, Langeveld may not have been aware of the text. Heidegger too uses concrete examples in his phenomenological reflections on the nature of The Thing. What is a thing? Heidegger explains that one needs “nearness” to understand the “thingness of the thing.” Next, Heidegger (1971) abruptly announces as follows: “The Jug is a thing” (p. 166). In other words, Heidegger does not bother to say, let’s take the jug as an example—yet that is exactly what he does. He then continues with an extensive descriptive explication of the phenomenological features of The Jug. And, of course, he uses the example of The Jug to address the meaning of The Thing. This essay is an exceptionally fine example of Heidegger’s way of “doing phenomenology on the things” (pun intended). True, some parts of the features of “The Jug as the Thing” are so exhaustively detailed that the reader may sometimes feel like being sucked into an ontological whirlwind, as one tries to stay attentive to the subtle and profound distinctions of Heidegger’s descriptive explications of the ontic eidos of the thing:

When we fill the jug, the pouring that fills it flows into the empty jug. The emptiness, the void, is what does the vessel’s holding. The empty space, this nothing of the jug, is what the jug is as the holding vessel. . . . But if the holding is done by the jug’s void, then the potter who forms sides and bottom on his wheel does not, strictly speaking, make the jug. He only shapes the clay. No—he shapes the void. . . . The vessel’s thingness does not lie at all in the material of which it consists, but in the void that holds. (Heidegger, 1971, p. 169)

The point of this passage is that we cannot understand what a “thing” is, in Heidegger’s special sense of the word, employing the properties of an object, as a substance with properties, or even the mental representation as “occurrent” (*vorhanden*). Rather, by taking the jug as an example, Heidegger shows how the thing things (shows its essence). He uses the example to show that the meaning of the jug lies, in part, in the relationality that the jug establishes between the person and the earth (how it nourishes thirst in its fullness) and between people (the host and guest). In the pouring, people experience the generosity (or skimpiness) of the jug. The notion that the potter merely “shapes the void” draws attention to the peculiar “passivity” that Heidegger takes to be essential to human productivity.

Now, Langeveld too utilizes examples in “The Thing in the World of the Child.” Yet his examples orient to make explicit and clarify understandings of a child’s unique bodied, relational, and temporal existence within the world. Consider his example of the gift of the tiny feather of a sparrow:

Consider the four-year-old child who comes to her mother, who is busy with the newborn baby, and has a “treasure” in her hand. It is the tiny feather of a sparrow. “This is for little brother, because he is still so small.” Now that is a true gift! It is not *le petit cadeau qui soutient l’amitie* [the little present that supports friendship] but rather, here we see *l’amour qui soutient les petits cadeaux* [love that supports small gifts]. This feather is a sign of a union of love. The feather is small—so be it: Is not the little brother small too? But how delicate and soft the feather is! It almost makes the beholder delicate and soft as well. (Langeveld in van Manen & van Manen, 2021, p. 129)

Here is the phenomenological insight: “Whoever gives a gift . . . gives him- or herself.” The thing in itself is more than its tangibility, for it bears the “symbol of the love of the giver.” Methodologically, the present (*cadeau*) is an eidetic for the gift (but the present gives little more than its trivial objectedness). So, the feather as a thing is more than a mere object because it is a thing that things, in Heidegger’s sense. But Langeveld does not approach the meaning of things in the manner of Heidegger, although some of Langeveld’s explications bring out similar sensibilities. For example, the seesaw is a thing that requires two young people to join each other. It is a thing of physical and human togetherness, a communion.

When Langeveld explores the meaning of the gift and the present, he does so by giving an example of a pedagogical situation. A situational analysis makes it possible for Langeveld to show how the phenomenon of a *gift* differs from the phenomenon of a *present*. But, of course, a more radical analysis of the gift and gift-giving is possible. Jacques Derrida is a master of aporias, showing how our ordinary intentions and actions involve paradoxes, insoluble contradictions, and impasses. He calls these predicaments undecidables. For example, when we are hospitable or give someone a gift, we may think we are doing so with no strings attached. We are giving this gift out of the goodness of our hearts. But Derrida (1995) shows how things are not so simple. The conditions of possibility are at the same time the conditions of impossibility. We cannot give a gift without receiving something in return, if only gratitude or the inner satisfaction we have done good. Gifts create debts. So, is it really possible to give a gift? Or is gift-giving ultimately always some kind of exchange? It seems indeed as if authentic giving or hospitality is an undecidable: neither possible nor impossible. Here, we see how phenomenology makes possible rich interpretive explications and understandings that may not only be undecidables but also incommensurables when perceived in contrasting existential contexts.

As mentioned, Langeveld offers phenomenological interpretations of other things—the filled and empty cardboard box, the ball, a special pen, plates, and cups, to name a few—to show how a child’s sensemaking transcends the physical properties of things. The child comes to know freedom from a carefree being in the world with things, and yet the thing-world also presents the experience of limitation:

One plays in the sand, the water and the snow; one builds with stones, always because they invite one to do so. The flowing water, the swirling sand, and the snow that sticks together well, they speak a totally pathic language and entice us toward play.

But soon the unspecified manipulation of formless materials must stop, and organization begins. Whoever wants to build something out of sand or snow or wants to put a bridge over a stream must come to reckon with the objective qualities of sand, snow, and water. Again, the thing-world lures the organizing, the sense-making person, just as one is lured into a magic forest. (Langeveld in van Manen & van Manen, 2021, p. 138)

The difference between Heidegger’s example of the jug and Langeveld’s example of things such as the slipper or the feather is that the jug is a thing that already has a history of meanings for Heidegger, meanings that are existentially embedded in the world in which humans are born. So, in everyday life, seeing a jug is not a matter of sensemaking, of wondering what this thing is as it presents itself, but rather the presence of the jug is a matter of reaching for it to pour some of its contents. Or one might use the jug to hold some cut flowers. In our everyday world, it is actually rather odd to consider the jug as an example of the phenomenology of a thing. Only when inspired by the phenomenological attitude would one consider the profound inceptual meanings that Heidegger is able to intuitively “see” and explicate in his pathic and vocative language and style about the nature of this thing, the jug.

In contrast, when Husserl gives the example of looking at a thing, he does not consider any particular thing but rather things in general—when he gives his foundational description of the phenomenon of adumbration. When we look at a thing, such as a book, every perception of consciousness is perspectival. We never see the whole thing (book) at once but what is visible is, first of all, a surface: “I see it now from this ‘side,’ now from that, continuously perceiving it from ever differing sides” (Husserl, 1954/1970a, p. 157). And yet, we retain a sense-intuition of certainty about the thing. So, as we never perceive a thing from all sides at once, but only in adumbrated perspectives, we become phenomenologically aware that seeing some-thing empirically is not necessarily knowing it fully or completely.

So, if Husserl would see a thing like a jug, he would look at its surfaces from above, below, and the inside, but would he know it like Heidegger knows a jug? Husserl is interested in how the thing “appears” and “shows itself” as we look at its perceptual surfaces and dimensions. But, of course, when we see this book, we also may see its invitation to be read, or the obligation that we need to return the book to its lender, and so on. Heidegger is interested in how the jug appears as a meaningful thing in our existential world. Now, in contrast, in the world of the child, things do not have dimensions or meanings yet, but the child can climb over things, throw things, sit inside things (like in a big box), and thus, the child learns to make sense of the meaning of things in an as yet open world.

Langeveld’s exploration of the thing in the world of the child reveals a different modality of sense. If the child sees a jug on the kitchen table, he or she may curiously look inside or even put or hide something inside it. Similarly, Langeveld shows that when the child sees a slipper, it can be many different things. This imaginative or playful seeing is more a matter of the child’s existence of open sensemaking that may or may not be restrained by some of the limitations that Langeveld supposes could be part of the way that the thing in the world of the child appears. For example, the slippers are mama’s and so the slippers give themselves as the things that the mother puts on her feet. The sense the child makes is that the slippers embody “mama.” So, Langeveld gives the example of the slipper to show how in the child’s world, the slipper is not merely an object worn on the foot. The slipper becomes sensual as it finds its way into a child’s mouth, or handy as it is employed to hammer some objects, or cradle-like as it becomes a bed for a doll. The slipper as a thing invites playful sensemaking for the child.

Things do more: through the encounter with things, children develop a past with each other. The things bring them together. Ultimately, Langeveld offers originary and nuanced reflections on “The Thing in the World of the Child.” We gain insights that are immanent to the thinging of things. We catch phenomenological glimpses and sights into the lifeworld of the child that give the adult thoughtful understandings that make possible pedagogically tactful interactions of the adult with the child.

## Being Guided by the Phenomenological Attitude

As attentive readers, we may have been aware of the phenomenological attitude that Langeveld adopts in his phenomenological analysis of the thing in the child’s world. This attitude functions as a method of attentiveness. Langeveld (in)famously pointed out that in Husserl’s writings, the term “phenomenology” occurs in two meaning contexts: “to signify a method and to signify a philosophy.” Langeveld chose to use the term primarily to refer to the method and remain impartial to Husserl’s development of a phenomenological philosophy (Langeveld, 1972). Of course, Langeveld was correct that phenomenology is the name of a method. We suggest that the third stream of publications, that Kockelmans describes, were guided by the method of the phenomenological attitude to gain insights into the originary meanings of a phenomenon like the thing in the world of the child. In other words, the phenomenological attitude functions like a rich and internalized method. And yet, a feature of Langeveld and his colleagues’ works appears to be that they rarely engaged in arguing or articulating the philosophical technicalities of phenomenology for their inquiry. This absence of theorizing about methodological and philosophical issues was likely because these proponents were all professional practitioners, often with significant clinical responsibilities. While it is evident that most of these proponents had read the philosophical phenomenological literature, apparently, they just were not that interested in philosophizing about the conditions of doing phenomenology. However, their disinterest for theorizing was also a consequence of their view of the nature of phenomenology: “the phenomenologist is obsessed by the concrete . . . he distrusts theoretical and objective observations,” said van den Berg (1972), in his *A Different Existence* (p. 76).

One might ask, “How were these individuals able to practice phenomenology in their respective fields of psychology, medicine, pedagogy, law, and psychiatry when they generally opted not to engage in exegetical studies of the philosophical discourses of Husserl and his followers that established phenomenology?” The point is that through their familiarity with the works of Husserl, Stein, Heidegger, Scheler, Sartre, and Merleau-Ponty, the Utrecht phenomenologists internalized the phenomenological attitude while largely ignoring the technical philosophical discourses that preoccupied the increasing number of academic philosophers who were engaged in exegetically arguing about abstracted phenomenological themes, issues, systems, and theories of their time.

How can the internalization of the phenomenological attitude be described? We have to begin with the “natural attitude” that we all carry most of the time. Dermot Moran (2013) pointed out that the natural or naturalistic attitude is so taken-for-granted that the bearers of this attitude do not know that they have it. In contrast, phenomenologists must understand the nature of this natural attitude, and they must understand the critical and methodological importance of transforming the natural attitude into the phenomenological attitude that enables phenomenological seeing and intuition.

The significance of the phenomenological attitude is evident already in the foundational explications of phenomenology by Heidegger (1927/1962), Merleau-Ponty (1962), Henry (1990/2008), and others. Heidegger (1972/1975) stated that Husserl’s teaching took the form of practicing phenomenological “seeing” (p. 78). Merleau-Ponty (1962) described phenomenology as a “manner or style of thinking” (p. viii). In addition, Henry (1990/2008) put that the “transcendental possibility of experience is the original phenomenalizing of the phenomenality of the phenomenon” (p. 104), which is opening the path to the meaning of a phenomenon. None of these methodological characterizations refer to the application of a technical or scientific set of procedural steps. The practice of phenomenological “seeing” is an internalized, perception-based, and sensitive serendipitous act. Furthermore, the methods of the epoché and the reduction are involved, in a broad sense, as the distinguishing critical feature and essence of the phenomenological attitude.

While Husserl (1954/1970a) characterized the practice of the epoché and the reduction in many different ways (he mentions the transcendental, phenomenological, skeptical, vocational, and psychological epoché and reduction), a key feature is that it makes possible a transformation of the natural attitude. It is hard to fully realize and recognize the depth, pervasiveness, and taken-for-grantedness of the objectivism, naturalism, positivism, and shallow distractionism that shapes our way of looking at ourselves and the world around us and how this has affected the ecology of the planet and human civilizations. Even expressing our naturalistic predicament like this betrays a blindness to the fact that we always already immediately see the things around us as objects and objective forces.

Etymologically, the term “attitude” refers to the disposedness, disposition, posture, and fittedness of the comportment of a certain way of seeing, feeling, and acting, according to the online Oxford English Dictionary. An attitude regarding an object of thought can be deliberately or even unwittingly adopted. And an attitude can also be purposefully altered or disposed. This is a key idea for Husserl’s phenomenology as it is the taken-for-grantedness of the natural attitude that prevents us from seeing the so-called hidden meanings of phenomena. He defines an “attitude” (*Einstellung*) as

a habitually fixed style of willing life comprising directions of the will or interests that are prescribed by this style, comprising the ultimate ends, the cultural accomplishments whose total style is thereby determined . . . Humanity always lives under some attitude or other. (Husserl, 1954/1970a, p. 280)

Husserl speaks about “the natural primordial attitude, of the attitude of original natural life” (p. 281) as the attitude of the culture and the historical age in which we are born, and that forms the default natural orientation to life that characterizes our being-in-the-world.

Langeveld published in 1972, *Capita from the General Methodology of Pedagogical Science*, in which he addressed the issue of method in his phenomenological work. He suggested that one can debate Husserl (1954/1970a) on philosophical issues and he criticized the assumptions introduced by Husserl’s elaborations of transcendental subjectivity. Husserl had proposed how the knowing self must experimentally annul the existence of the world, meaning annul the self as the concrete subject of this knowledge of the world, by experimentally pretending that there is no world and no knowing subject. In Langeveld’s view, this sense of transcendental subjectivity abrogates the empirical “I” and the lived “world” relative to objective knowing. Langeveld asked rhetorically what the point would be of a phenomenological philosophy that only yields forms of knowing and understanding that are so detached from everyday human experience that they fail to serve the existential lives of (professional) practitioners or any other human beings? What good are transcendental truths and “pure” ideas that can neither be related to the concrete world nor to the lives of those who live in this world?

Langeveld suggested that refusing to follow Husserl into this philosophy of transcendental subjectivity did not mean that one must give up on the phenomenological method of inquiry. To reiterate, he pointed out that in Husserl’s writings, the term “phenomenology” occurs in two general meaning contexts: “to signify a method and to signify a philosophy.” Langeveld (1972) chose to use the term primarily to refer to the method and remain impartial to Husserl’s development of a phenomenological philosophy. Thus, Langeveld and his colleagues mostly were interested in foundational and epistemological issues to the extent that they contributed to doing phenomenological research and writing on the phenomena. Yet they certainly shared an understanding of the philosophical method that lies at the core of phenomenology. This understanding was realized through the sensibility of what may be called the “phenomenological attitude or disposition.”

Langeveld and his colleagues adopted (wittingly or unwittingly) the phenomenological attitude as a tacit application or transformation of the epoché and the reduction in a broad sense. While many contemporary phenomenologists no longer mention the Husserlian terminology of the epoché and the reduction, they nevertheless seem to adopt through a process of mimesis, the methods of the epoché and the reduction when practicing phenomenology on concrete phenomena. In contrast, while some more theoretically inclined philosophers may understand the necessity of adopting a phenomenological way of seeing, they yet may fail to do so as they are too preoccupied arguing about technicalities. It is hard for them to let go of the exegetical attitude. In other words, a scholar may be able to expertly traverse and address numerous thematic topics and issues in texts by Husserl and other foundational phenomenologists and yet may strangely lack the talent or ability to adopt the phenomenological attitude required to write an insightful phenomenological study on a concrete phenomenon or event.

In Husserl’s texts, we seldom meet extended concrete examples of the practice of the phenomenological attitude. Dermot Moran (2000) said that, although Husserl’s project was ostensibly “*descriptive* phenomenology,” ironically, Husserl’s writings are often abstract, focusing on technicalities, and notoriously “lacking in concrete examples” (p. 63). In an interview, van den Berg also remarked that Husserl remained too tied to his desk and hardly moved outside the philosophical world (Kruger, 1985). It is well-known that even Husserl’s home was an extension of his university office when he invited students for philosophy seminars. Indeed, it might be interesting to speculate how Husserl’s followers might have been inspired and how the development of phenomenology might have unfolded in a richer fashion if Husserl himself had indulged some of the time to focus on concrete and down-to-earth lifeworld phenomena in his pursuit of a pure phenomenology.

Langeveld, Buytendijk, Linschoten, and van den Berg admired Husserl’s genius and his dedication and perfectionism and they recognized his need for continuously rewriting his articles. Still, despite this admiration, they did not think they needed to follow Husserl into all those explorations of the foundational technicalities that Husserl obstinately kept pursuing and that keeps present-day philosophers still obsessed and preoccupied. Thus, we also hope that more philosophically based authors may recognize the value and join the effort to do more phenomenology on the concrete phenomena of our professional and everyday existence.

## Approaching Phenomenology as the Study of Examples

Buytendijk once referred to phenomenology as the “science of examples” (van Manen, 2014, p. 257). Whether taking the form of vignettes, anecdotes, or narratives, “examples” may be understood as rhetorical and aesthetic devices for evoking phenomenological understandings or phenomenological knowledge that cannot necessarily be expressed, explained, or explicated in a straightforward propositional or prosaic manner. The use of “phenomenological examples” is a clear feature of phenomenological texts that focus on the phenomena themselves. Examples in this methodical sense are also found in the wider phenomenological philosophical literature: the example of “boredom” while waiting for the train in the study of metaphysics in Martin Heidegger (1995, p. 93), the example of the myth of “the Gaze of Orpheus” in the study of writing in Maurice Blanchot (1981, pp. 99–104), the example of the voyeur looking through the keyhole of the door in “the look” in Jean-Paul Sartre (1956, pp, 259, 260), “Homer’s Odysseus” as an example of *The Homecomer* in Alfred Schutz (1971, pp. 106–119), the example of “Morpheus” in *The Fall of Sleep* in Jean-Luc Nancy (2007, pp. 8, 9), and so on.

Although Husserl rarely used detailed reflections of concrete examples to analyze and explicate the meaning of a concrete phenomenon or event, a well-known use of example occurs when Husserl describes the cogito as an act. He says,

Let us start with an example. In front of me, in the dim light, lies this white paper. I see it, touch it. This perceptual seeing and touching of the paper as the full concrete experience *of* the paper that lies here as given in truth precisely with these qualities, precisely with this relative lack of clearness, with this imperfect definition, appearing to me from this particular angle—is a *cogitatio*, a conscious experience. (Husserl, 1913/2014, p. 65)

Husserl sets himself the task of describing the phenomenon of conscious experience (*Erlebnis*), meaning “lived experience.” According to Husserl, the cogitatio, the stream-of-consciousness lived experience, in the fullness of its unity, can be seen to give access to the essence of every lived experience.

The Eidos, the *pure essence*, can be exemplified intuitively in the data of experience, data of perception, memory, and so forth, but just as readily *also in the mere data of fancy (Phantasie)*. Hence with the aim of grasping an essence itself in its *primordial* form, we can set out from corresponding empirical intuitions, *but we can also set out just as well from non-empirical intuitions, intuitions that do not apprehend sensory existence, intuitions rather of a merely imaginative order*. (Husserl, 1913/2014, p. 14)

The philosopher Edward Casey (2000, 2007) has written several insightful and eloquent phenomenological studies on topics such as places and landscapes, the glance, and imagining. Casey (2000) asserts that the phenomenological method as conceived by Husserl takes its beginning from carefully selected examples. Note earlier that W. R. Boyce Gibson’s translation of Husserl’s *Ideas* reads as follows:

The Eidos, the *pure essence*, can be exemplified intuitively in the data of experience. (Husserl, 1913/2014, p. 14)

Casey, however, translates this passage as follows:

The eidos or pure essence, can be exhibited by example. (Casey, 2000, p. 23)

With this slight but pronounced modification, Casey lets Husserl make his point even more clearly and emphatically than Husserl probably meant himself. But the point for us is that phenomenology may indeed be seen to proceed through examples. For Casey, the “example” is not only the method to carefully select his studies, but he also uses the notion of “example” as a methodological device. In his study, *Imagining*, Casey (2000) takes his own experiences as a source for constructing narrative examples to investigate the meaning of the phenomenon “imagining.” Furthermore, he affirms that it is not only fictional texts that can function as examples but also observed and fictive objects, events, and actions.

Phenomenological method takes objects, events, or acts—whether real or imagined—as exemplifying an essence or essential structure. In this way their basic constitution is made perspicuous, and examples become the specific vehicles or privileged media of eidetic insights. (Casey, 2000, p. 24)

Casey wants to make the strong case that examples that exhibit an essence or essential structure with a maximum of evidential lucidity can achieve eidetic insights. Even carefully selected factual or empirical material may serve as phenomenological examples, but only after they have been fictionalized through the application or performance of the reduction (Husserl, 1913/1983).

It is important to keep in mind that phenomenology does not deal with facts. Accordingly, we may need to allow that some examples only partially serve the purpose of the phenomenological reduction; while they present evidentially perspicacious examples, they may remain linguistically ambiguous or enigmatic. For the Utrecht phenomenologists, the methodological power of the “example” also serves an analytic purpose. The “example” does not express what one knows through argument or conceptual explication, but, in a vocative manner, an “example” lets one *experience* what one does not know. There is an indirectness in the turn to the narrative meaningfulness of phenomenological examples (see also van Manen, 2014).

The example can make the singular experienceable and thus knowable as an *indite* method of phenomenological writing. While the methods of the epoché and the reduction are engaged in an attempt to gain insights into the originary meaning of a phenomenon, it is the indite methods, the vocative aspects of writing, that assist in bringing phenomenological insights to textual understanding. The online Oxford English Dictionary defines the term “indite” in this way: “to put into words, compose (a poem, tale, speech, etc.); to give a literary or rhetorical form to (words, an address); to express or describe in a literary composition.” We use indite here to focus on the semiotic or writing practices that present the linguistic, methodological dimension to phenomenological thinking, inquiring, and writing. An “example” often takes shape as a story (as in existential literary fiction) and thus orients to the singular. Indeed, any literary story or novel is always some unique narrative that brings out the particularity or singularity of a certain phenomenon, event, or life.

In the exegetical phenomenological literature, little attention appears to be paid to the methodological significance of the “example” in phenomenological writing. But, some of the leading phenomenologists commonly speak of and reach for an “example” when examining a phenomenon or event for its phenomenal features. Unfortunately, many of Husserl’s (1913/1983) “examples” are seemingly overly simple, such as a reference to seeing a tree, in his explication of the noema and intentionality (pp. 214, 215). But Husserl’s (1964) most famous and extended “example” is probably contained in his study of time consciousness. In his description of our inner consciousness of time, Husserl uses the example of hearing a familiar melody. In hearing a well-known musical melody, the present notes of the melody and the notes just past are retained in retention while the notes about to be heard are already anticipated as protention. Thus, Husserl explicates and shows the streaming structure of ongoing retentions and protentions as primal impressional consciousness in the exemplary experience of hearing a familiar melody.

Similarly, when Heidegger (2001) reflects on the meaning of the “thing,” he uses the example of a jug. When Henry (1988/2009) presents the aesthetic revelation of the invisible essence of “life,” he uses the paintings of Wassily Kandinsky as an example. When Sartre (1956) discusses the experience of “negation and nothingness,” he says that he needs an example, and he describes having an appointment with Pierre in the café where they are supposed to meet at 4 o’clock (p. 9). But as he arrives at the café and looks around, Sartre discovers, “He is not here.” Next, Sartre (1956) explores how it is that we “see” this absence that is a nothing (a not-being-there) and yet not a nothing (the absence of not being there) (1956, p. 10). Interestingly, all of these aforementioned examples have acquired iconic fame in the phenomenological literature. They have become classic or well-known phenomenological anecdotes, vignettes, narratives, or images, and it matters not whether they are fictional, imagined, or real, in an empirical or biographic sense.

In contrast, in the traditional and qualitative social sciences, examples are usually employed as concrete or illustrative “cases-in-point” to clarify an abstract idea or theory. This commonly used form of example-as-case-in-point is meant to make theoretical knowledge more accessible, concrete, or intelligible, although the example itself may not contribute to the knowledge. Indeed, examples are often used as informative illustrations. But, an example-as-illustration can be left out of the text without compromising the text. So, it is essential to realize that “phenomenological examples” differ radically from such explanatory, clarifying, or illustrative uses of examples. The phenomenological notion of “example” is methodologically a unique semiotic figure for phenomenological inquiry.

Examples in phenomenological texts have evidential significance because the example is the example of something experientially knowable or understandable that is not directly expressible—it is a universal singularity. If a singularity were to be expressed in ordinary prose, it would immediately vanish. Why? Because language cannot really express a singularity by naming or describing it. A singularity cannot be grasped directly through words because words are already generalized bits of language. Language universalizes. However, and this is paradoxical, the “phenomenological example” as a story can provide access to the phenomenon in its universal singularity. It makes the “singular” knowable and understandable. Every fictional story or novel has at its core a singularity: a unique theme or signification.

The etymology of the Greek word for model is to “show something in something and thus make it present” in an interpretive methodical sense. Günther Figal makes special use of the term “model” as an equivalent term for “example.” He says, “a model is a definitive example” (Figal, 2010, p. 29). To reflect in a hermeneutic phenomenological manner on the meaning of something is to examine it as an originary model. The model is like an incept (as opposed to a concept). It points toward the originary meaning of something. Some models are more appropriate or better suited to get at the originary meaning of something. And so, models (as examples) must be well-chosen because the essence of the matter has to be expressed in the model. In the words of Figal (2010), “models are supposed to be distinguished by their pregnancy; they must prove themselves as such by really letting something be shown in them” (p. 30).

Similarly, Giorgio Agamben (2002) uses the term “example” interchangeably with paradigm: “example” means *para-deigma*. Agamben says, “paradigm means simply ‘example’ . . . a single phenomenon, a singularity.” A singularity is, by definition, single and unique—it does not share properties in common with anything else. In other words, a singularity has no specifiable identity (idem); it has no recognizable sameness except that it is self-same. A singularity is only identical to itself (ipseity). Interestingly, Agamben (1995) points out that a true example is neither particular nor universal.

To reiterate, it would be wrong to assume that the “example” in phenomenological inquiry is used as an illustration in an argument, or as a particular instance of a general idea, or as an empirical datum from which to develop a conceptual or theoretical understanding. Instead, the phenomenological example is a philological device that holds in a certain tension the intelligibility of the singular. How can the example do this? It can do this because the example mediates our intuitive (self-evidential) grasp of a singularity, which is precisely the project of phenomenology. Again, we need to sense the paradoxicality of this explication of a critical methodological aspect of phenomenological inquiry, thinking, and writing.

The singularity of the singular may show itself by way of the example. “The example lets the singular be seen,” says Agamben (1995, p. 10). But one could perhaps equally say that the phenomenological example reconciles the incommensurable couplet of the particular and the universal. In other words, singularity emerges in the deconstructive fusion of the particular with the universal. In this sense, the phenomenological example expresses the singular as universal. So, the example is somewhat of an enigma and contradiction. This idea may be seen as a phenomenological variation on Georg Wilhelm Friedrich Hegel’s notion that a lived experience originates as particularity but becomes recognizable as universal.

Previously, we showed how Langeveld used the story of the feather as an example of a child’s gift. Spiegelberg (1972) speaks of these anecdotes as “colorful vignettes that are characteristic of Dutch phenomenology” (p. 87). In *Doing Phenomenology*, Spiegelberg employs vignette-style expressions to provide concrete contexts for his phenomenological analysis of the phenomenon of *approval*. Joseph Kockelmans, too, observes how the phenomenologists of the Utrecht School frequently make use of poetry and literature. He sees three reasons: First, many “great poets and novelists have seen something very important and have spoken of it in a remarkably adequate way” that is useful for phenomenological explication. Second, phenomenologists may use literary sources “to illustrate a point on which the phenomenologists wish to focus attention.” And third, most important, “poetic language . . . is able to refer beyond the realm of what can be said ‘clearly and distinctly’” (Kockelmans, 1987, pp. viii, ix).

Experiential descriptions, in the form of colorful vignettes, should not be taken as mere embellishing or illustrative examples of points made in a text. We must avoid confusing phenomenological examples as if they are mere didactical explanations. Rather, these narrative stories should be approached as fictional vignettes or narrative anecdotes or aesthetic and poetic objects. Phenomenology reflects on “examples” to discover what is originary, singular, or essential about a phenomenon or event. The example is the presencing of something experientially knowable or understandable that is not easily directly expressible—a singularity or an essence. In other words, the “phenomenological example” as fictionalized story provides access to the eidetic meaning of the phenomenon in its singularity. It makes the essence as the “singular” knowable and understandable.

We have pointed out that the example is indeed a way that phenomenology may proceed. Buytendijk, Spiegelberg, Kockelmans, Casey, Figal, and Agamben have made clear, in different ways, that the example is a powerful methodological device to reveal eidetic and intentional phenomenological meaning. Skilled and eloquent phenomenologists perfected the use of concrete “examples” to evoke understandings inherent in concrete but phenomenologically universal narrative descriptions, gained from or modeled on fictional, poetic, mythological, and aesthetic sources.

## Putting Phenomenology Back Into Phenomenology

We have highlighted that the phenomenological attitude and the use of examples are two key methodological features for doing phenomenology on phenomena as described by Kockelmans and Spiegelberg. As Kockelmans indicated, these phenomenological studies done directly on the phenomena or concrete things are the type of phenomenological inquiries intended by the founding scholars like Husserl, Heidegger, Merleau-Ponty, and other original phenomenological thinkers. We are indeed struck by the uncanny observations made by Herbert Spiegelberg who, in his later years, found that so much philosophical scholarship of phenomenology lacks the vitality of what phenomenology could be. So, our aim is to try to put phenomenology back into phenomenology by showing how this had been practiced by the Utrecht proponents and how it may inspire our present-day and future phenomenological research projects; see *Classic Writings for a Phenomenology of Practice* (van Manen & van Manen, 2021). We propose that these classic writings demonstrate a way of doing phenomenology directly on the “phenomena” or on the “things” themselves. We also propose that these publications are guided by a phenomenological attitude aimed to arrive at meaningful insights, sensitive to concrete experience, and proceeding through phenomenological examples.
